# Point-wise correlations between 10-2 Humphrey visual field and OCT data in open angle glaucoma

**DOI:** 10.1038/s41433-020-0989-7

**Published:** 2020-06-01

**Authors:** Paola Cirafici, Guido Maiello, Chiara Ancona, Alessandro Masala, Carlo Enrico Traverso, Michele Iester

**Affiliations:** 1 IRCCS Ospedale Policlinico San Martino, Genoa, Italy; 2 Clinica Oculistica, DiNOGMI, University of Genoa, Genoa, Italy; 3 Department of Experimental Psychology, University of Giessen, Giessen, Germany

**Keywords:** Outcomes research, Optic nerve diseases

## Abstract

**Purpose:**

Optical Coherence Tomography (OCT) is a powerful instrument for helping clinicians detect and monitor glaucoma. The aim of this study was to provide a detailed mapping of the relationships between visual field (VF) sensitivities and measures of retinal structure provided by a commercial Spectral Domain (SD)-OCT system (RTvue-100 Optovue).

**Methods:**

Sixty-three eyes of open angle glaucoma patients (17 males, 16 females, and mean age 71 ± 7.5 years) were included in this retrospective, observational clinical study. Thickness values for superior and inferior retina, as well as average values, were recorded for the full retina, the outer retina, the ganglion cell complex, and the peripapillary retinal nerve fiber layer (RNFL). RNFL thickness was further evaluated along eight separate sectors (temporal lower, temporal upper, superior temporal, superior nasal, nasal upper, nasal lower, inferior nasal, and inferior temporal). Point-wise correlations were then computed between each of these OCT measures and the visual sensitivities at all VF locations assessed via Humphrey 10-2 and 24-2 perimetry. Lastly, OCT data were fit to VF data to predict glaucoma stage.

**Results:**

The relationship between retinal thickness and visual sensitivities reflects the known topography of the retina. Spatial correlation patterns between visual sensitivities and RNFL thickness along different sectors broadly agree with previously hypothesized structure–function maps, yet suggest that structure–function maps still require more precise characterizations. Given these relationships, we find that OCT data can predict glaucoma stage.

**Conclusion:**

Ganglion cell complex and RNFL thickness measurements are highlighted as the most promising candidate metrics for glaucoma detection and monitoring.

## Introduction

Glaucoma is an optic neuropathy characterized by death of retinal ganglion cells and visual field (VF) loss. Optical Coherence Tomography (OCT) is a powerful instrument for helping clinicians to detect and monitor glaucoma onset and progression. However, the diagnostic capacity of OCT data to predict specific spatial patterns of glaucomatous visual loss, particularly in central vision, is unclear. To address this gap in the literature, here we determine the point-wise correlations between VF sensitivities and retinal thickness measurements taken via spectral domain OCT (SD-OCT) in open angle glaucoma patients. Specifically, we measured the thickness of the full retina, the outer retina, and the ganglion cell complex (GCC) at the macula, as well as the thickness of the peripapillary retinal nerve fiber layer (RNFL). We related these retinal thickness measurements to Humphrey 10-2 and 24-2 VF measurements. In particular, both macular GCC thickness and RNFL thickness have been demonstrated to have diagnostic potential for detecting early, moderate, and severe glaucoma [1, 2]. Yet how these measurements relate to specific patterns of VF loss has yet to be determined. If OCT retinal thickness measurements well relate to glaucomatous VF loss, it should further be possible to derive glaucoma stage, typically assessed through perimetry, directly from OCT data.

With respect to the RNFL, Garway-Heath et al. were the first to provide a retinotopic map of the optic nerve head (ONH) [3]. From this mapping it was immediately apparent that in standard perimetry some retinal areas are oversampled or under sampled with respect to the RNFL distribution. In the fovea-centered 24-2 grid of standard automated perimetry (24-2 VF), 68 test points are located regularly at 6° from each other. Garway-Heath et al. noted that the direction of the RNFL or the proportion of axons within an RNFL cross-section in each sector was not regularly distributed across VF test points, and this was due to the distribution of the fibers around the ONH. The precise effects of the arrangement of VF test points on the assessment of the structure–function correlation between nerve fiber damage and VF loss are yet to be established, although previous reports have shown that the detection rate of glaucomatous VF defects is affected by the arrangement [4] and number [5–7] of test points.

In addition to these structure–function considerations, it is also important to note that glaucoma is generally a bilateral disease, the severity of which may be asymmetric in the two eyes [8]. Therefore, the aims of the current study are: (1) to assess the degree to which glaucomatous deficits in one eye predict the probability of similar deficits in the other eye [9] (2) to provide a detailed mapping of the relationships between VF sensitivities and several measures of retinal structure provided by a commercial Spectral Domain (SD)-OCT system (RTvue-100 Optovue), (3) to test whether these OCT data can be employed to predict behaviorally assessed glaucoma stage. Understanding the relationship between both macular GCC and RNFL with corresponding central visual function will help to better tailor strategies to detect early glaucomatous damage and disease worsening.

## Methods

### Subjects

Thirty-three open angle glaucoma patients were included for this retrospective observational study and evaluated at the University Eye Clinic of Genoa. Data collection procedures were approved by the institutional review board of the Ospedale Policlinico San Martino IRCSS, Genoa. The study was conducted in accordance with the tenets of the Declaration of Helsinki.

#### Inclusion and exclusion criteria

Main inclusion criteria were age greater than 40 years and a diagnosis of open angle glaucoma (monolateral or bilateral) under medical treatment. These glaucoma patients performed two 24-2 and two 10-2 perimetry tests per year on a Humphrey Field Analyzer II-i. The most recent two reliable VF examinations were considered for the study.

Exclusion criteria were: secondary glaucoma; abnormalities of the anterior segment of the eye; cornea abnormalities with entities that could affect IOP evaluation; comorbid conditions distinct from glaucoma that could cause perimetric defects (degenerative myopia, cataract, and maculopathy); best corrected visual acuity < 2/10; any previous ocular surgery except for cataract extraction; pregnancy or breast-feeding.

All study participants underwent a comprehensive ophthalmic examination, which included: best correct visual acuity; macular and peripapillary RNFL thickness by SD-OCT RTvue-100 Optovue. Thickness values for superior and inferior retina, as well as average values, were recorded for the full retina, the outer retina, the GCC, and the peripapillary RNFL. The peripapillary RNFL thickness was further evaluated along eight separate sectors (temporal lower, temporal upper, superior temporal, superior nasal, nasal upper, nasal lower, inferior nasal, and inferior temporal).

### Data analyses

The Pearson correlation coefficient was employed to assess the degree of linear dependency between any two variables. Where possible, OCT measures and VF data were collected from both eyes of each patient. To assess the degree to which OCT and VF measurements in one eye were related to the same measurements in the other eye, we correlated all data from patients’ left and right eyes. As expected, we found that the data from patients’ left and right eyes were not statistically independent. To account for these between-eye correlations [9], for all subsequent analyses the data from patients’ left and right eyes was averaged. On these averaged data, point-wise correlations were then computed between each of the OCT measures and the visual sensitivities at all VF locations assessed via Humphrey 24-2 and 10-2 VF. Outliers were removed prior to each individual analysis; outliers were identified as any values that departed from the mean by more than three times the standard deviation. Means and confidence intervals for correlation coefficients were estimated using Fisher’s Z transform to ensure variance stabilization [10].

To assess whether OCT data can be employed to predict glaucoma stage, we first determined glaucoma stage following the Enhanced Glaucoma Staging System 2 (GSS 2) [11]. Corrected Pattern Standard Deviation (CPSD) were derived from PSD following the simple conversion: CPSD = PSD − 0.7058 [11]. Next, we fit OCT data to MD and CPSD values. Since OCT parameters are most likely correlated, we performed principal component analysis on the OCT data to obtain a set of uncorrelated OCT-derived parameters. Multiple linear regression models were constructed to relate these decorrelated OCT predictors to MD and CPSD values from both 24-2 and 10-2 VF data. Parameters were added to the models using a stepwise inclusion procedure, beginning from the most basic models that included only intercept terms. Parameters were added to the model one at a time and were retained only if the Bayesian Information Criterion decreased by more than 2 units. We estimated glaucoma stage from these fitted MD and CPSD values. We used simple correlation to compare original and OCT-derived glaucoma stage estimates.

Throughout all analyses, values of *p* < 0.05 were considered statistically significant. Given our sample size, our analyses could detect a medium to large effect sizes of *r* = 0.4. This choice helped ensure that any statistically significant results we report are likely to correspond to clinically significant effects as well.

## Results

Thirty-three open angle glaucoma patients (17 males and 16 females) were included in the study. Both eyes were considered except in three cases in which only one eye was used. Thirty patients were classified as primary open angle glaucoma, two as pseudo-exfoliation glaucoma and one as pigmentary glaucoma.

### Interocular correlations

**Fig. 1:**
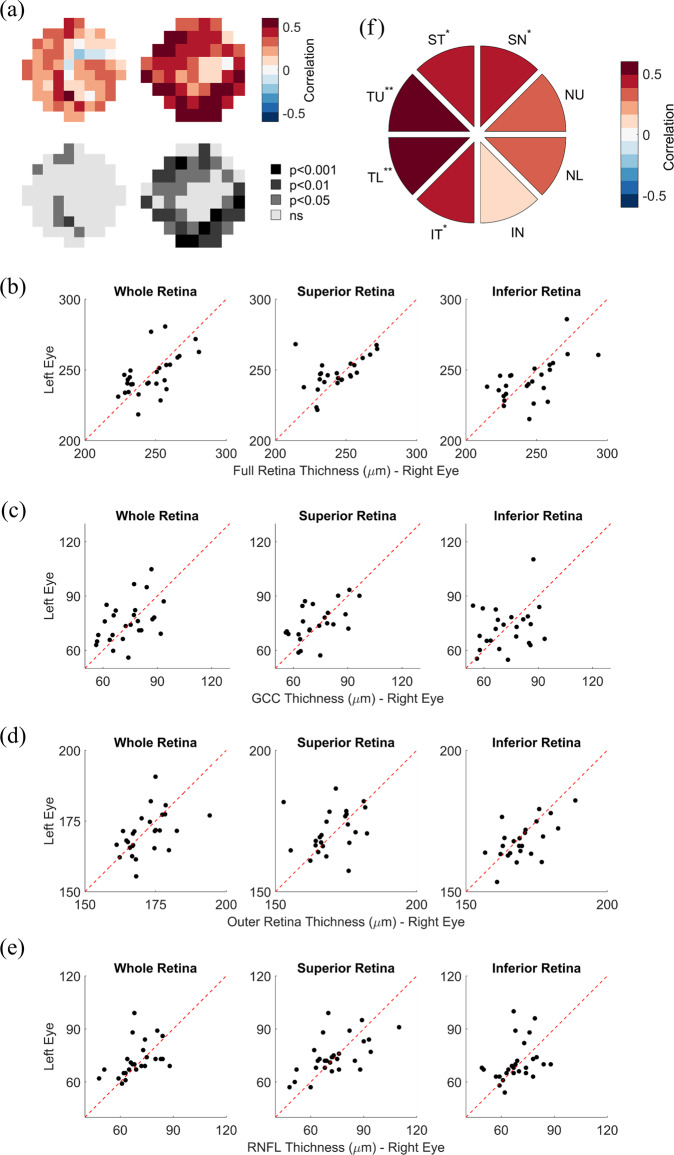
Interocular correlations. **a** Correlations between visual field sensitivity of left and right eyes of study participants, for both 10-2 (left) and 24-2 (right) visual field data. The top half of the panel shows the strength of the correlation at each tested visual field location, color-coded as in the legend. The bottom half of the panel shows the level of statistical significance of the correlations at each tested visual field location. **b–e** Scatter plots plotting the retinal thickness of left eyes against the retinal thickness of right. The different panels show the data for the thickness of (**b**) the full retina, (**c**) the GCC, (**d**) the outer retina, and (**e**) the RNFL. In each panel, from left to right, the subplots show retinal thickness data averaged across the whole retina, the superior retina, or the inferior retina. **f** Correlations between the thickness of the RNFL of left and right eyes at the different RNFL sectors. The strength of the correlation at each sector is color-coded as shown by the legend. *p < 0.05; **p < 0.01.

Figure 1 shows the degree to which glaucomatous deficits in one eye predicted the probability of similar deficits in the other eye. Figure 1(a) shows that foveal and parafoveal 10-2 VF sensitivities measured in the right eye only weakly correlated with visual sensitivity measured in the left eye, with an average correlation coefficient across VF locations of *r* = 0.21, 95% confidence interval [0.18–0.25]. Conversely, peripheral 24-2 visual sensitivities measured in the right eye were more strongly and significantly correlated with visual sensitivities measured in the left eye; mean *r* = 0.42, 95% confidence interval [0.38–0.46].

Figure 1b–e shows that the thicknesses of various layers of the retina in one eye were strongly related to the thicknesses of the retinal layers of the other eye. The thickness of all retinal layers combined (Fig. 1b) was strongly correlated across the two eyes, independently of whether the measurement was taken across the whole retina (*r* = 0.59, *p* = 0.0010), or across only the superior (*r* = 0.51, *p* = 0.0089) or inferior (r = 0.59, *p* = 0.0019) portions of the retina.

The thickness of the GCC (Fig. 1c) was also significantly correlated across the two eyes when measured across the whole (*r* = 0.45, *p* = 0.020) and the superior (*r* = 0.54, *p* = 0.0063) retina, but not for the inferior retina (*r* = 0.24, *p* = 0.25). The thickness of the outer retina (Fig. 1d) was significantly correlated across the two eyes when measured across the whole (*r* = 0.48, *p* = 0.011) and the inferior (*r* = 0.61, *p* = 0.0017) retina, but not for the superior retina (*r* = 0.21, *p* = 0.31). The thickness of the RNFL (Fig. 1e) was significantly correlated across the two eyes if measured across the whole (*r* = 0.44, *p* = 0.022) or the superior (*r* = 0.59, *p* = 0.00091) retina, but not when measured across the inferior (*r* = 0.35, *p* = 0.076) portions of the retina. In addition, Fig. 1f shows that the thickness of the RNFL was significantly correlated across the two eyes for five out of eight RNFL sectors.

### Point-wise correlations between Humphrey visual field and OCT data

**Fig. 2:**
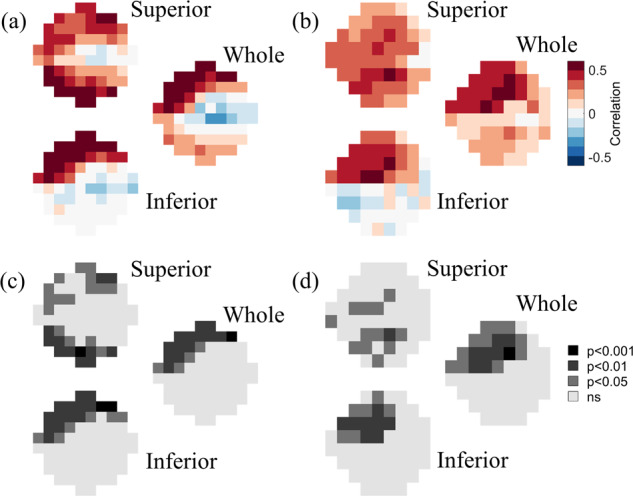
Full retinal thickness correlations. Point-wise correlations between full retinal thickness versus (**a**, **c**) 10-2 and (**b**, **d**) 24-2 visual field data. **a**, **b** show the strength of the correlation at each tested visual field location, color-coded as shown by the legend. **c**, **d** show the level of statistical significance of the correlations at each tested visual field location. In each of the four panels, the top, bottom, and middle-right images represent the correlation maps between visual field data and the full retinal thickness of the superior retina, the inferior retina, and the whole retina, respectively.

The full thickness of all retinal layers was significantly and positively correlated with both 10-2 and 24-2 VF data (Fig. 2). Specifically, Fig. 2a, c shows that, near the fovea, full retinal thickness values measured across the whole and the inferior retina were most strongly and significantly correlated with the superior 10-2 VF sensitivities (peak correlations: *r* = 0.61, *p* = 0.00043 and *r* = 0.63, *p* = 0.00038), whereas retinal thickness values measured across the superior retina were most strongly correlated with inferior 10-2 VF sensitivities (peak *r* = 0.61, *p* = 0.00075). This pattern of spatial correlations was also similarly observed in the peripheral VF (Fig. 2b, d). Full retinal thickness values measured across the whole and the inferior retina were most strongly correlated with the superior 24-2 VF sensitivities (peak correlations: *r* = 0.57, *p* = 0.00089 and *r* = 0.53, *p* = 0.0028), retinal thickness values measured across the superior retina were most strongly correlated with inferior 24-2 VF sensitivities (peak *r* = 0.55, *p* = 0.0018).

**Fig. 3:**
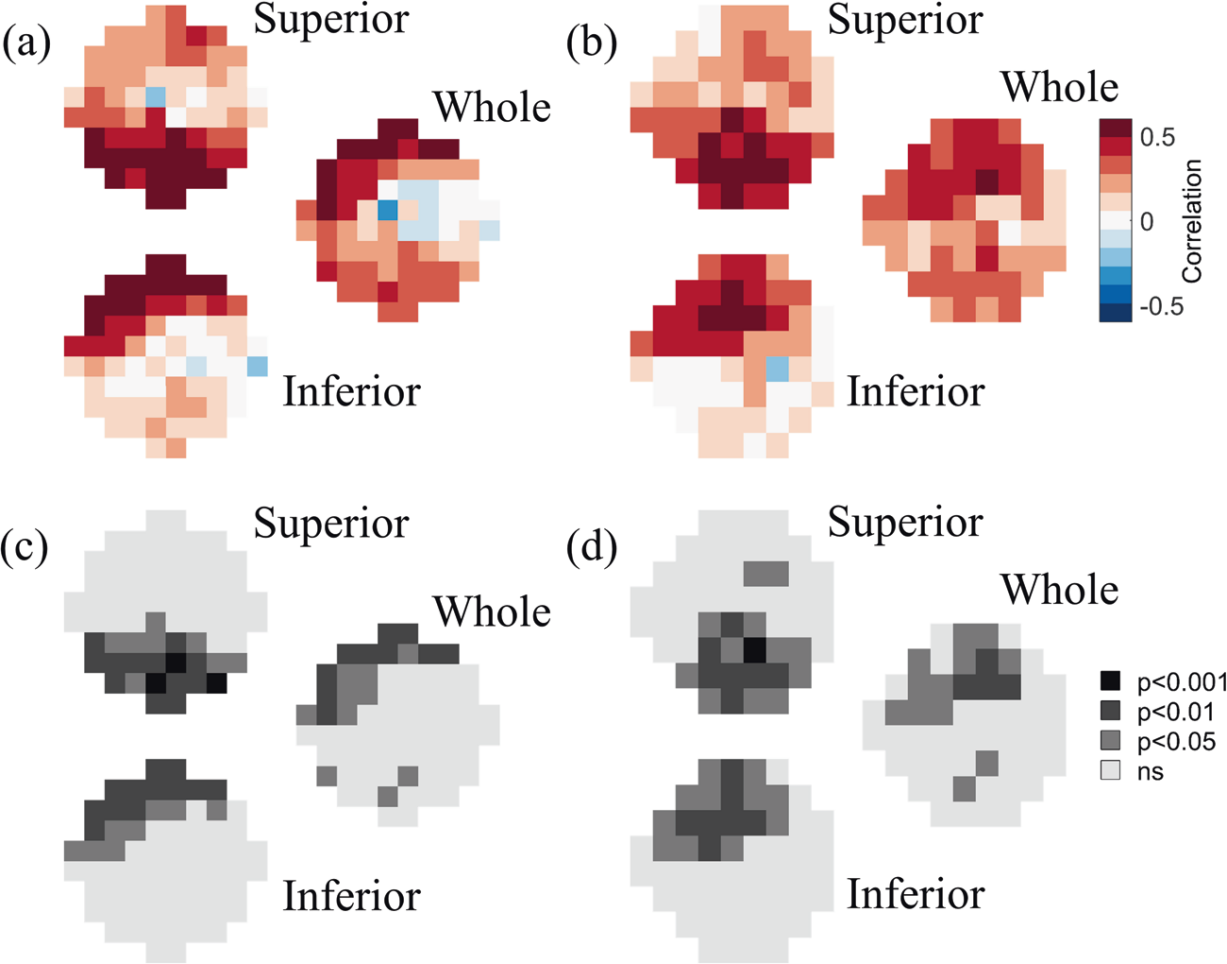
GCC correlations. Point-wise correlations between GCC thickness versus (**a**, **c**) 10-2 and (**b**, **d**) 24-2 visual field data. **a**, **b** show the strength of the correlation at each tested visual field location, color-coded as shown by the legend. **c**, **d** show the level of statistical significance of the correlations at each tested visual field location. In each of the four panels, the top, bottom, and middle-right images represent the correlation maps between visual field data and the GCC thickness of the superior retina, the inferior retina, and the whole retina, respectively.

The patterns of spatial correlations between GCC thickness and VF data (Fig. 3) were strikingly similar to the patterns observed for the full retinal thickness. Near the fovea (Fig. 3a, c), GCC thickness values measured across the whole and the inferior retina were significantly correlated with superior 10-2 VF sensitivities (peaks: *r* = 0.58, *p* = 0.0012 and *r* = 0.58, *p* = 0.0016), whereas GCC thickness values measured across the superior retina were most strongly correlated with inferior 10-2 VF sensitivities (peak *r* = 0.64, *p* = 0.00049). In the peripheral VF (Fig. 3b, d), GCC retinal thickness values measured across the whole and the inferior retina were most strongly correlated with the superior 24-2 VF sensitivities (peaks: *r* = 0.55, *p* = 0.0018 and *r* = 0.52, *p* = 0.0040), GCC thickness values measured across the superior retina were most strongly correlated with inferior 24-2 VF sensitivities (peak *r* = 0.60, *p* = 0.00067).

**Fig. 4:**
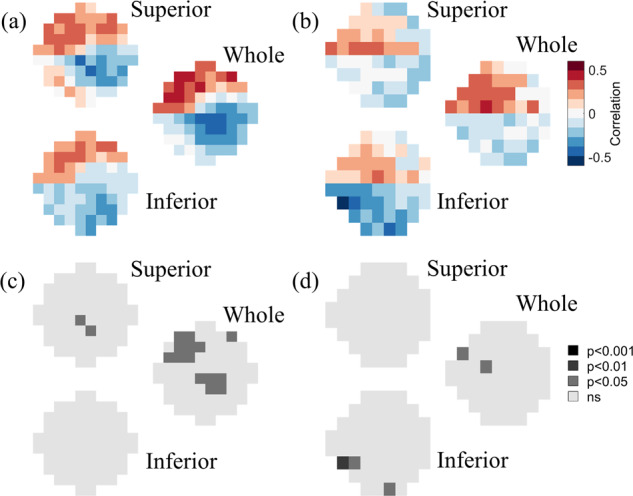
Outer retina thickness correlations. Point-wise correlations between outer retina thickness versus (**a**, **c**) 10-2 and (**b**, **d**) 24-2 visual field data. **a**, **b** show the strength of the correlation at each tested visual field location, color-coded as shown by the legend. **c**, **d** show the level of statistical significance of the correlations at each tested visual field location. In each of the four panels, the top, bottom, and middle-right images represent the correlation maps between visual field data and the outer retina thickness of the superior retina, the inferior retina, and the whole retina, respectively.

The thickness of the outer retina was generally less strongly (and often inversely) associated with VF sensitivity (Fig. 4). Near the fovea (Fig. 4a, c), outer retina thickness values measured across the whole, superior, or inferior retina were positively correlated to superior 10-2 VF sensitivities and negatively correlated to inferior sensitivities (peak correlations: *r* = −0.47, *p* = 0.011; *r* = −0.48, *p* = 0.012; *r* = −0.34, *p* = 0.080). The pattern was similar in the peripheral VF as well (Fig. 4b, d; peak correlations: *r* = −0.42, *p* = 0.022; *r* = 35, *p* = 0.064; *r* = −0.51, *p* = 0.0048).

**Fig. 5:**
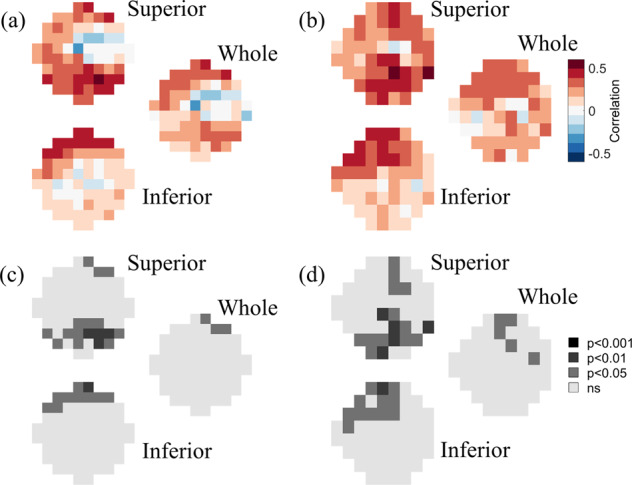
RNFL correlations. Point-wise correlations between RNFL thickness versus (**a**, **c**) 10-2 and (**b**, **d**) 24-2 visual field data. **a**, **b** show the strength of the correlation at each tested visual field location, color-coded as shown by the legend. **c**, **d** show the level of statistical significance of the correlations at each tested visual field location. In each of the four panels, the top, bottom, and middle-right images represent the correlation maps between visual field data and the RNFL thickness of the superior retina, the inferior retina, and the whole retina, respectively.

The patterns of spatial correlations between RNFL thickness and VF data (Fig. 5) were once again similar to the patterns observed for the full retinal thickness and for GCC thickness. Near the fovea (Fig. 3a, c), RNFL thickness values measured across the whole and the inferior retina were significantly correlated with superior 10-2 VF sensitivities (peaks: *r* = 0.42, *p* = 0.020 and *r* = 0.48, *p* = 0.0088), whereas RNFL thickness values measured across the superior retina were most strongly correlated with inferior 10-2 VF sensitivities (peak *r* = 0.50, *p* = 0.0054). In the peripheral VF (Fig. 3b, d), RNFL retinal thickness values measured across the whole and the inferior retina were most strongly correlated with the superior 24-2 VF sensitivities (peaks: *r* = 0.38, *p* = 0.031 and *r* = 0.47, *p* = 0.0083), RNFL thickness values measured across the superior retina were most strongly correlated with inferior 24-2 VF sensitivities (peak *r* = 0.50, *p* = 0.0033).

**Fig. 6:**
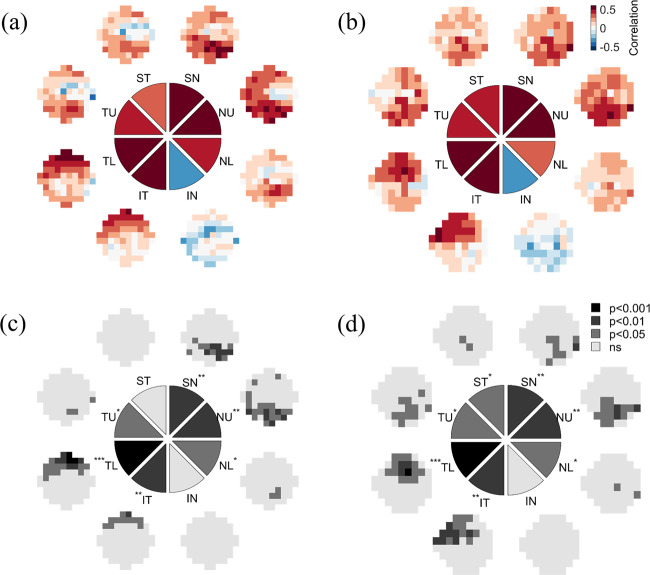
Sectorial RNFL correlations. Point-wise correlations between RNFL thickness along eight separate sectors versus (**a**, **c**) 10-2 and (**b**, **d**) 24-2 visual field data. **a**, **b** show the strength of the correlation at each tested visual field location, color-coded as shown by the legend, for each of the 8 RNFL sectors as indicated by the central disc. The color of the wedges represents the maximum correlation observed across all visual field locations for each individual sector. **c**, **d** show the level of statistical significance of the correlations at each tested visual field location. *p < 0.05; **p < 0.01; ***p < 0.001.

Figure 6 shows the patterns of spatial correlations between RNFL thickness measured along eight separate sectors and VF sensitivity. Near the fovea (Fig. 6a, c), RNFL thickness measured along inferior temporal and temporal lower sectors significantly correlated with upper VF sensitivities (peaks: *r* = 0.50, *p* = 0.0070; *r* = 0.63, *p* = 0.00030), RNFL thickness along the temporal upper, superior nasal, nasal upper, and nasal lower sectors significantly correlated with the lower VF sensitivities (peaks: *r* = 0.39, *p* = 0.034; *r* = 0.58, *p* = 0.0012; *r* = 0.53, *p* = 0.0040; *r* = 0.42, *p* = 0.026), while the correlations between VF sensitivities and RNFL thickness along superior temporal and inferior nasal sectors did not reach statistical significance (peaks: *r* = 0.35, *p* = 0.070; *r* = −0.36, *p* = 0.062). In the visual periphery (Fig. 6b, d) RNFL thickness measured along inferior temporal and temporal lower sectors significantly correlated with upper VF sensitivities (peaks: *r* = 0.54, *p* = 0.0022; *r* = 0.58, *p* = 0.00080), RNFL thickness along the temporal upper, superior temporal, superior nasal, nasal upper and nasal lower sectors significantly correlated with the lower VF sensitivities (peaks: *r* = 0.44, *p* = 0.016; *r* = 0.41, *p* = 0.025; *r* = 0.50, *p* = 0.0053; *r* = 0.50, *p* = 0.0053; *r* = 0.38, *p* = 0.039), and the correlations between VF sensitivities and RNFL thickness along the inferior nasal sector did not reach statistical significance (peak *r* = −0.32, *p* = 0.083).

### Deriving glaucoma stage from OCT data

**Fig. 7:**
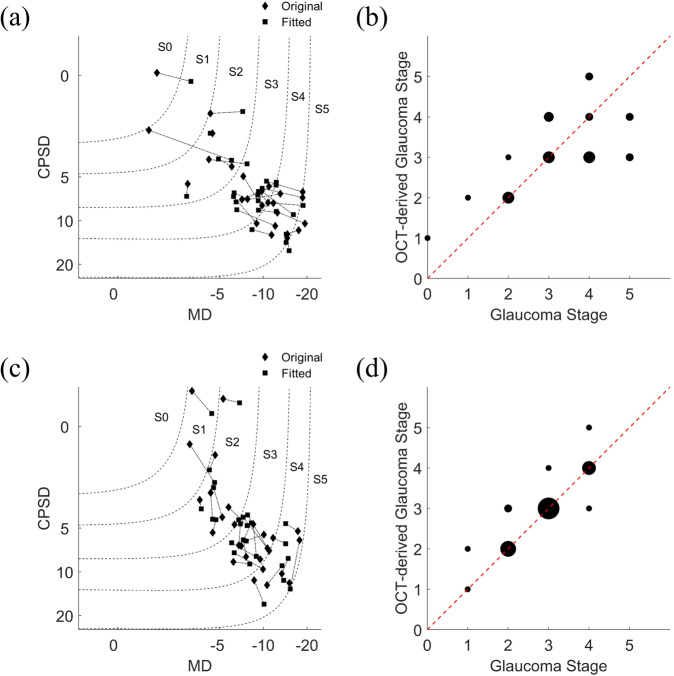
Deriving glaucoma stage from OCT data. Deriving glaucoma stage from OCT data. **a** Original (diamonds) and OCT-fitted (squares) MD and CPSD data placed in the GSS 2 chart for 24-2 perimetry. Continuous lines connect original and fitted data values from individual participants. Dotted lines show boundaries between glaucoma stages 0 through 5. **b** OCT-derived glaucoma stage estimates are plotted against glaucoma stage derived from 24-2 perimetry. Dot size is proportional to the number of occurrences for each data point. (**c, d**) As (**a,** **b**), except for 10-2 perimetry.

Figure 7 shows the results of our procedure for deriving glaucoma stage directly from OCT data. Figure 7a shows original and fitted MD and CPSD data from 24-2 perimetry placed in the GSS 2 chart. Stepwise regression models were able to significantly predict 24-2 MD (*F*_1,21_ = 5.23, *p* = 0.0044, *r*^2^ = 0.50) and CPSD (*F*_1,14_ = 10.00, *p* = 7.6 × 10^−5^, *r*^2^ = 0.89) data from OCT parameters. Figure 7b shows that glaucoma stage derived from fitted MD and CPSD parameters well correlates to glaucoma stage determined through 24-2 perimetry (*r* = 0.68, *p* = 0.00012, *r*^2^ = 0.47). Figure 7c shows original and fitted MD and CPSD data from 10-2 perimetry placed in the GSS 2 chart. Stepwise regression models were able to significantly predict 10-2 MD (*F*_1,19_ = 8.71, *p* = 8.2 × 10^−5^, *r*^2^ = 0.76) and CPSD (*F*_1,17_ = 7.82, *p* = 0.0001.6, *r*^2^ = 0.81) data from OCT parameters. Figure 7c shows that glaucoma stage derived from fitted MD and CPSD parameters correlates to glaucoma stage determined through 10-2 perimetry even more strongly than for 24-2 perimetry (*r* = 0.87, *p* = 2.5 × 10^−9^, *r*^2^ = 0.77).

## Discussion

RNFL thickness can be assessed at the ONH, in the peripapillary area and in the macula and all have been shown to be good indicators of retinal ganglion cell damage for glaucoma diagnosis [12, 13]. In particular, it has become increasingly common to assess glaucomatous damage at the macula [14–20].

Hood et al. first showed that macular damage occurs in about 90% of patients with early glaucoma, and macular damage within 10° is increasingly recognized as a common disease feature [14–17]. These central scotomas could be both diffuse and focal [15] and could interfere with patients’ vision more than typical and well-known nasal step or arcuate defects which are located more peripherally. Recently, some authors have even suggested that both structural and functional measures of macular damage are associated with decreases in vision-related quality of life in glaucoma patients [21, 22].

Using a Retinal Thickness Analyzer (Talia Technology Ltd, Neve Ilan, Isel) Zeimer et al. first hypothesized the possibility to detect the loss of ganglion cells analysing the posterior pole [23]. Following the introduction of time-domain OCT, Greenfield et al. observed a reduction of macular thickness in early- and moderate-stage glaucoma by using time-domain OCT and demonstrated that these macular changes were correlated with VF sensitivity [13]. With the introduction in clinics and research of a newer generation of OCT, the spectral domain OCT (SD-OCT), the loss of ganglion cells has become easier to detect. Many authors have shown significant correlations between macular ganglion cell thickness and VF sensitivity and have also found that macular parameters exhibit higher and more reproducible correlations with visual function than peripapillary parameters [13–17, 24–28].

In our study, we found that the zones with lower correlations with retinal thickness measurements were located more peripherally in the VF and this could be due to the fact that the peripheral field is more prone to fluctuation or variability. The use of the 10-2 VF perimetry for central VF testing more accurately represents visual function in the macula. The use of 24-2 VF testing could therefore be insufficient to accurately characterize central VF deficits due to its limited spatial sampling. Indeed, the distance between testing locations in the 24-2 perimetry is 6°, whereas testing locations are 2° apart in 10-2 perimetry. While 24-2 perimetry is useful in detecting peripheral glaucomatous damage such as nasal step or arcuate scotomas, 10-2 perimetry is likely more sensitive for the detection of paracentral scotomas. Our results further show that paracentral scotomas are also more likely to be asymmetric across the two eyes compared with peripheral VF defects. This may impact binocular vision and stereoscopic depth perception, and particularly impede fine eye movements in depth and eye-hand coordination [29–31].

Our results further show that GCC and RNFL thickness measurements are more sensitive than outer retina thickness measurements in predicting VF loss. This confirms that in glaucoma the main changes occur in the inner layers of the retina where ganglion cells are present. For both 10-2 and 24-2 perimetry, correlation patters were strongest between OCT measurements of the inferior retina and superior VF sensitivities, and for OCT measurements of the superior retina and inferior VF sensitivities. These correlations could be affected by the selection of the cohort of patients and could be due to the type of damage found. Of course, the more common structural damage is in the infero-temporal disc rim/RNFL outlining the obtained results. When the patterns of spatial correlations between VF sensitivity and RNFL thickness measured along eight separate sectors was analyzed, we further observed significant structure–function correlations for most of the sectors. As in previous studies [32], 10-2 VF perimetry provided higher accuracy in defining the structure–function relationship in glaucoma. Given the tight and nuanced nature of the relationship between retinal thickness and visual function, we find that OCT data can even be employed to directly predict behaviorally assessed glaucoma stage [11].

In conclusion the structure–function maps we obtained were largely consistent with the mappings previously reported. The inferior retina (measured via OCT) was found to be a more vulnerable area and was well correlated to superior central VF test points. Collected data and analyses highlight GCC and RNFL thickness measurements as the most promising candidate metrics for glaucoma detection and monitoring.

## Summary

### What was known before

- Structure–function maps with 30-2 visual field have already been published, but less is known on 10-2 visual field.

### What this study adds

- This study shows ganglion cell complex and peripapillary correlations as first step to create a new 10-2 clinical map.
